# Poor health-related quality of life despite Lupus Low Disease Activity State or Definitions of Remission in systemic lupus erythematosus (SLE) remission in patients with SLE: results from a clinical trial setting

**DOI:** 10.1136/rmdopen-2025-006061

**Published:** 2025-10-31

**Authors:** Ioannis Parodis, Julius Lindblom, Leonardo Palazzo, Alexander Tsoi, Nursen Cetrez, Henri Ala, Mandana Nikpour, Adrian Levitsky, Vibeke Strand

**Affiliations:** 1 Division of Rheumatology, Department of Medicine Solna, Karolinska Institutet, Karolinska University Hospital, and Center for Molecular Medicine (CMM), Stockholm, Sweden; 2 Department of Rheumatology, Faculty of Medicine and Health, Örebro University, Örebro, Sweden; 3 University of Sydney School of Public Health, Sydney, New South Wales, Australia; 4 Department of Rheumatology, Royal Prince Alfred Hospital, Camperdown, New South Wales, Australia; 5 Division of Immunology/Rheumatology, Stanford University School of Medicine, Stanford, California, USA

**Keywords:** Systemic Lupus Erythematosus, Health-Related Quality Of Life, Fatigue, Pain

## Abstract

**Objective:**

To determine the prevalence of poor health-related quality of life (HRQoL) in patients with systemic lupus erythematosus (SLE) in Lupus Low Disease Activity State (LLDAS) or Definitions of Remission in SLE (DORIS) remission and sustained LLDAS or sustained DORIS remission, after a 52-week therapeutic intervention.

**Methods:**

We analysed data from four phase III trials of belimumab in SLE (BLISS-52, BLISS-76, BLISS-SC, EMBRACE; n=2406). Sustained LLDAS/remission was defined as persistent LLDAS/remission for at least two visits, maintained through week 52. Poor HRQoL was defined as Short Form-36 (SF-36) physical/mental component summary (PCS/MCS) and domain scores ≤the normative fifth percentile, Functional Assessment of Chronic Illness Therapy – Fatigue (FACIT-F) scores <30 and responses of ‘some/moderate problems’ or ‘extreme/major problems’ in any of the five dimensions of the three-level version of EuroQol 5-Dimension (EQ-5D) health questionnaire.

**Results:**

At week 52, among patients in LLDAS, remission, sustained LLDAS and sustained remission, 15.7%, 13.6%, 14.3% and 9.0% reported poor SF-36 PCS, and 12.5%, 11.4%, 12.9% and 14.0% reported poor SF-36 MCS scores, respectively. The highest frequencies were reported in the physical functioning domain (24.0%–26.3%), while 18.5%–26.2% reported FACIT-F scores 30. Among EQ-5D dimensions, pain/discomfort yielded the greatest frequencies of poor HRQoL experience (27.9%–28.7%). While significant improvements were observed among patients achieving the treatment goals in all HRQoL outcomes over the 52-week study period, PCS scores remained below population norms.

**Conclusions:**

Despite LLDAS or DORIS remission, notable proportions of SLE patients report poor HRQoL, indicating that current therapeutic goal definitions do not fully capture patients’ perspectives of health.

**WHAT IS ALREADY KNOWN ON THIS TOPIC**

- Patients with systemic lupus erythematosus (SLE) often report impaired health-related quality of life (HRQoL), even in the presence of a favourable clinical response to therapy.
- Achieving low disease activity, as defined by the Lupus Low Disease Activity State (LLDAS) criteria, and remission, as defined by the Definition of Remission in SLE (DORIS) criteria, are key treatment targets in SLE. However, these definitions do not incorporate patient-reported outcomes.

**WHAT THIS STUDY ADDS**

- This study demonstrated notable frequencies of persistently impaired HRQoL among patients with SLE who met LLDAS or DORIS remission criteria after a 52-week therapeutic intervention in a clinical trial setting, with similar findings among those who sustained these states.
- Although significant improvements in HRQoL were observed over the 52-week treatment period, scores in physical domains remained suboptimal compared with population norms.

**HOW THIS STUDY MIGHT AFFECT RESEARCH, PRACTICE OR POLICY**

- This post hoc analysis of phase III belimumab trials in SLE has important implications for clinical practice, highlighting the need to include patient-reported measures in disease monitoring along with current treatment targets. Composite indices that integrate patient-reported health experience alongside clinical measures of disease activity may prove useful in the future.

## Introduction

 Despite notable improvements in medical care over the past two decades, people with systemic lupus erythematosus (SLE) still experience substantial diminutions of health-related quality of life (HRQoL) compared with the general population and with patients with other chronic diseases.^1 2^ The advent of biological therapy has contributed to significant improvements in SLE patients’ HRQoL,^3^,^7^ and responders to treatment have been shown to report greater improvements than non-responders.^8^,^10^ However, even in the setting of adequate clinical response, a substantial proportion of SLE patients still report poor HRQoL outcomes.^11^

The treat-to-target approach has been increasingly used in SLE, with the treatment goals of achieving remission, and when not possible, low disease activity (LDA).^12 13^ The most commonly used sets of criteria for LDA and remission are the Lupus Low Disease Activity State (LLDAS)^14 15^ and the Definitions of Remission in SLE (DORIS) remission^16 17^ criteria, respectively. Both DORIS remission and LLDAS have been associated with decelerated organ damage,^18 19^ better HRQoL^10 20 21^ and reversal of the activation of key signalling pathways in SLE.^22^ However, despite the known discordance between physician-centred outcome measures and patients’ perceptions of disease status,^23^ as well as explicit recommendations from the Outcome Measures in Rheumatology SLE working group,^24 25^ these definitions of LDA and remission used in isolation do not incorporate patient-reported outcomes (PROs).

As per current knowledge, there is still a dearth of data regarding the prevalence of patients who still experience poor HRQoL despite achieving the therapeutic targets of LDA or remission. This framed the scope of the present investigation, which aimed to determine the prevalence of adverse HRQoL outcomes in patients with SLE who had attained LLDAS or DORIS remission and sustained LLDAS or sustained DORIS remission, after a 52-week long period on standard therapy (ST) plus belimumab or placebo within the frame of four phase III clinical trials. By focusing on LLDAS and DORIS remission, which represent stringent, state-based treatment targets with recognised links to long-term outcomes, we sought to quantify, across multiple PRO instruments, the residual burden of poor HRQoL within patient subgroups meeting the criteria for these targets and to benchmark findings against population norms.

## Methods

### Study population

 We conducted a post hoc analysis using data from four clinical trials, that is, BLISS-52 (intravenous belimumab; NCT00424476; n=865),^26^ BLISS-76 (intravenous belimumab; NCT00410384; n=819),^27^ BLISS-SC (subcutaneous (SC) belimumab; NCT01484496; n=836)^28^ and EMBRACE (intravenous belimumab in SLE patients of African ancestry; NCT01632241; n=448).^29^ All patients judged eligible to be included in the trials fulfilled the revised American College of Rheumatology (ACR) criteria for SLE,^30^ were adults, had an anti-nuclear antibody titre ≥1:80 and/or serum anti-double stranded (ds)DNA antibody level ≥30 IU/mL at screening and a Safety of Estrogens in Lupus National Assessment Systemic Lupus Erythematosus Disease Activity Index (SELENA-SLEDAI)^31^ score ≥6 (BLISS-52 and BLISS-76) or ≥8 (BLISS-SC, EMBRACE). All patients were on stable non-biological ST for ≥30 days before the baseline of the double-blinded phase. Progressive restrictions were imposed during the trial periods on concurrent medications, as well as glucocorticoid intake. Patients with severe active central nervous system involvement or severe active lupus nephritis were excluded. Patients were randomised to receive belimumab 1 mg/kg, belimumab 10 mg/kg or placebo on top of ST for 52 weeks in BLISS-52 and for 76 weeks in BLISS-76. In EMBRACE, patients received either belimumab 10 mg/kg or placebo on top of ST. Belimumab and placebo were administered intravenously at baseline, week 2, week 4 and thereafter every fourth week until week 48 in BLISS-52 and EMBRACE and until week 72 in BLISS-76. In BLISS-SC, patients received weekly doses of belimumab 200 mg or placebo administered subcutaneously in addition to ST. In the present post hoc analysis, we included patients receiving belimumab at approved dose (10 mg/kg intravenous monthly or 200 mg SC weekly) or placebo and excluded patients receiving belimumab 1 mg/kg intravenous monthly. We analysed the initial 52 weeks of follow-up across all four trials. We accessed data through the Clinical Study Data Request (CSDR) consortium on approval from GlaxoSmithKline (Uxbridge, UK).

Prior to enrolment, written informed consent was obtained from all study participants. The trial protocols were subject to review and approval by regional ethics review boards at all participating centres and adhered to the ethical principles of the Declaration of Helsinki. The protocol for the present post-hoc analysis was approved by the Swedish Ethical Review Authority (2019–05498).

### Poor HRQoL

Poor HRQoL was defined as Medical Outcome Study Short Form-36 (SF-36) scale^32^ scores equal to or lower than the normative fifth percentile, that is, the worst 5% of the scores reported from a US population-based control group individually matched with the study participants for age and sex, as previously described;^11 33^ Functional Assessment of Chronic Illness Therapy – Fatigue (FACIT-F)^34^ scores <30, which denote severe fatigue; and responses of ‘some/moderate problems’ or ‘extreme/major problems’ in any of the five dimensions of the three-level version of EuroQol 5-Dimension (EQ-5D) health questionnaire (EQ-5D-3L).^35^

SF-36 is a questionnaire used for assessment of HRQoL over the preceding 4 weeks. Computation of patients’ responses to 36 questions results in eight domains, each representing a distinct HRQoL aspect, that is, physical functioning (PF), role physical (RP), bodily pain (BP), general health (GH), social functioning (SF), vitality (VT), role emotional (RE) and mental health (MH). SF-36 domain scores were calculated according to the SF-36v2 manual^36^ and transformed to generate domain scores ranging from 0 to 100. Subsequently, the SF-36 domain scores were weighted into two summary scores, that is, the physical component summary (PCS) and mental component summary (MCS). The component summary scores are normalised, with a mean of 50 and a SD of 10. All domains contribute to the derivation of PCS and MCS, although with different weightings. PF, RP, BP and GH are weighted positively in PCS and negatively in MCS and are referred to as the physical aspects of SF-36. SF, VT, RE and MH are weighted positively in MCS and negatively in PCS and are referred to as the mental aspects of SF-36. Higher scores in SF-36 domains and component summaries are interpreted as better HRQoL perception.^32^ As suggested in the literature, we considered a 5-point improvement in SF-36 domain scores and a 2.5-point improvement in PCS and MCS scores as the minimal clinically important difference (MCID).^10 11 37^

FACIT-F is a survey that evaluates the level of fatigue over the preceding 7 days. Patient responses to the 13 items of FACIT-F are transformed into a score ranging from 0 (maximal fatigue) to 52 (minimal fatigue).^34^ Changes of ≥4.0 points in the FACIT-F score were considered the MCID.^10 38^

EQ-5D-3L consists of a visual analogue scale, intended to reflect overall health status, and a descriptive system comprising five questions, which reflect five corresponding HRQoL dimensions, that is, self-care, mobility, usual activities, pain/discomfort and anxiety/depression. Respondents may report no problems (level 1), some/moderate (level 2) or extreme/major problems (level 3) in each one of these dimensions. Combinations of patient responses in this descriptive system represent the patient’s self-reported health status, which is referred to as an EQ-5D profile.^35^ EQ-5D utility index scores are derived from these profiles using country-specific value sets that assign preference weights to each health state. The MCID was set at 0.04 points, as previously described.^10 39^

### Clinical definitions

The LLDAS definition^15^ requires a SLEDAI 2000 (SLEDAI-2K)^40^ score ≤4, no major organ activity or fever, no new activity in any descriptor since the previous assessment and SELENA-SLEDAI^31^ physician’s global assessment (PGA) ≤1 (on a scale 0–3) and allows a prednisone equivalent dose ≤7.5 mg/day and immunosuppressive or approved biological agents at standard doses.

The DORIS definition of remission^17^ requires a clinical SLEDAI-2K score 0 and a SELENA-SLEDAI PGA score <0.5 (on a scale 0–3) while allowing serological activity and use of low-dose glucocorticoids (prednisone equivalent ≤5 mg/day) and immunosuppressive or biological agents at standard doses.

Sustained LLDAS and sustained DORIS remission at week 52 were defined as persisting LLDAS or DORIS remission, respectively, present at ≥2 visits 4 weeks apart and maintained through week 52, reflecting the visit schedule of the trials and reducing the likelihood of capturing transient states, as operationalised in prior post-hoc analyses of the similar datasets.^41^

### Statistical analysis

Descriptive statistics are reported as numbers (percentage) or the mean±SD. In case of non-normal distributions, the median IQR is indicated. Comparisons of continuous variables between baseline and week 52 were performed using the Wilcoxon signed-rank test. For comparisons between unrelated groups, the non-parametrical Mann-Whitney *U* test was used for continuous variables and the Pearson’s *χ*^2^ or Fisher’s exact test for binomial variables, as appropriate.

Statistically significant differences were considered those yielding *p* values <0.05. Analyses were performed with R Statistical Software V.4.4.2. (R Foundation for Statistical Computing, Vienna, Austria).

## Results

In total, 2409 patients formed the study population. Patient demographics and clinical data are reported in online supplemental table S1.

### Notable proportions of SLE patients experience poor HRQoL despite LLDAS or DORIS remission

**Figure 1:**
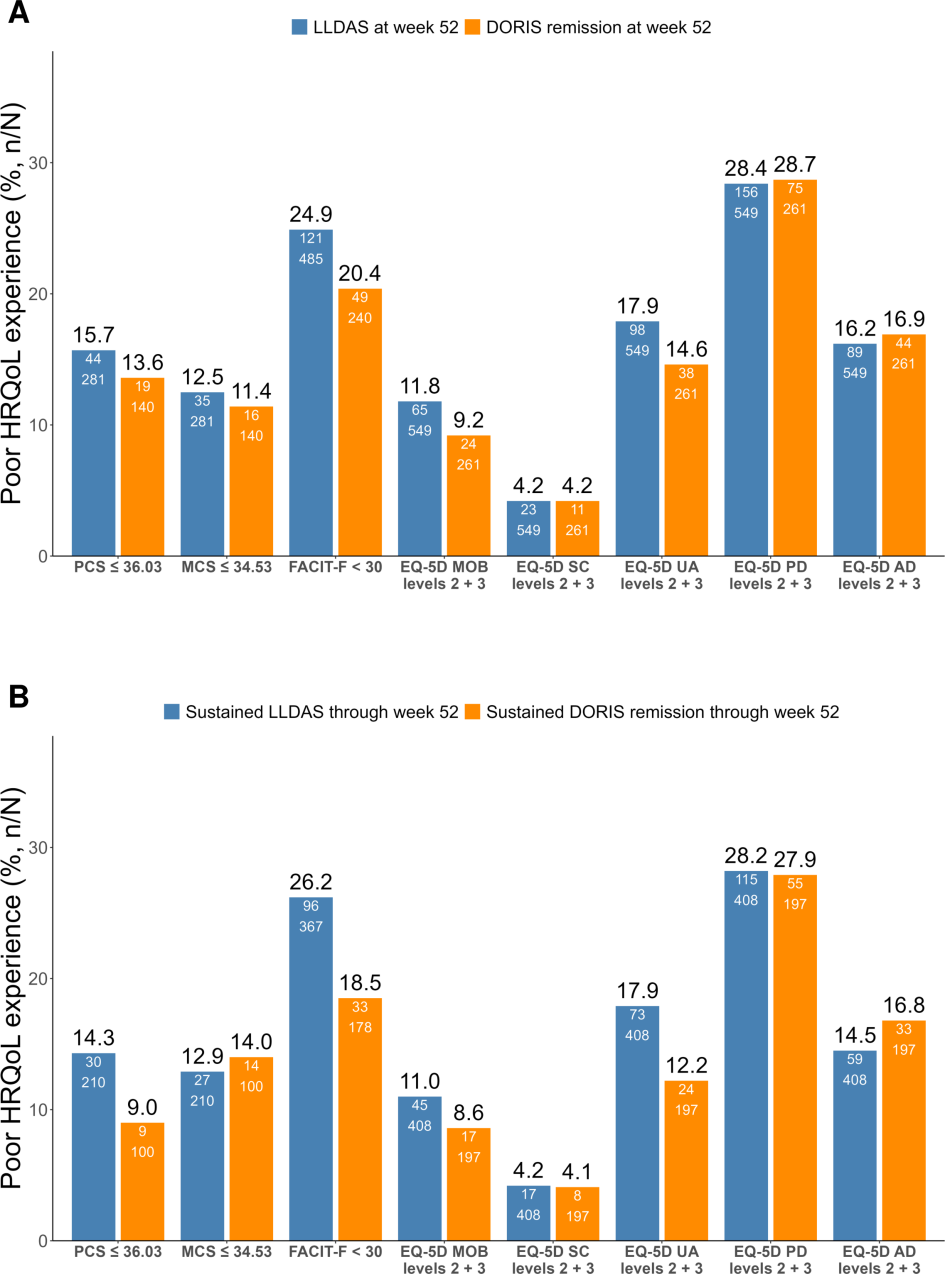
HRQoL outcomes among patients in LLDAS or DORIS remission. The bar plots illustrate the proportion of patients with poor HRQoL outcomes among those in LLDAS (blue bars) or DORIS remission (orange bars) at week 52 (A) and among those achieving sustained LLDAS (blue bars) or sustained DORIS remission (orange bars) (B). AD, anxiety/depression; DORIS, Definition of Remission in SLE; EQ-5D, EuroQol 5-Dimension health questionnaire; FACIT-F: Functional Assessment of Chronic Illness Therapy – Fatigue; HRQoL, health-related quality of life; LLDAS, Lupus Low Disease Activity State; MCS, mental component summary; MOB, mobility; PCS, physical component summary; PD, pain/discomfort; SC, self-care; SLE, systemic lupus erythematosus; UA, usual activities.

**Figure 2:**
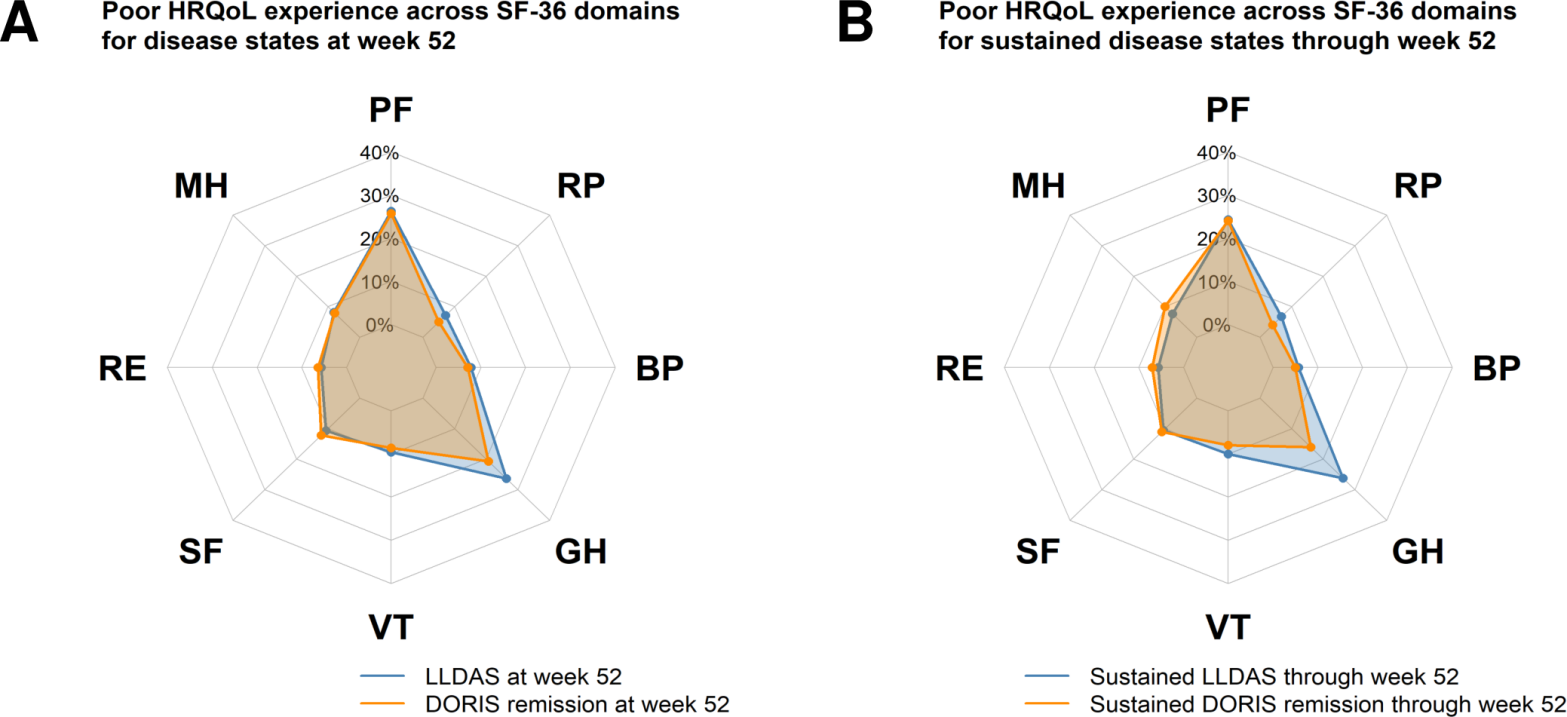
HRQoL outcomes among patients in LLDAS or DORIS remission. The radar (spider) charts illustrate mean SF-36 domain scores among patients in LLDAS (blue polygon) or DORIS remission (orange polygon) at week 52 (A) and patients in sustained LLDAS (blue polygon) or sustained DORIS remission (orange polygon; B). BP, bodily pain; DORIS, Definition of Remission in SLE; GH, general health; HRQoL, health-related quality of life; LLDAS, Lupus Low Disease Activity State; MH, mental health; PF, physical functioning; RE, role emotional; RP, role physical; SF, social functioning; SF-36, short form 36 health survey; SLE, systemic lupus erythematosus; VT, vitality.

As illustrated in figure 1 and detailed in online supplemental table S2, among patients attaining LLDAS at week 52, 44/281 (15.7%) reported poor SF-36 PCS scores, and 35/281 (12.5%) reported poor SF-36 MCS scores. Similarly, among patients attaining DORIS remission, 19/140 (13.6%) reported poor SF-36 PCS and 16/140 (11.4%) reported poor SF-36 MCS (figure 1A). Among patients attaining sustained LLDAS and sustained DORIS remission, 30/210 (14.3%) and 27/210 (12.9%) reported poor SF-36 PCS, and 9/100 (9.0%) and 14/100 (14.0%) reported poor SF-36 MCS (figure 1B). The greatest percentages of poor HRQoL were reported in the PF domain (24.0%–26.3%), followed by the GH domain (16.0%–26.3%) (figure 2A,B and online supplemental table S2).

At week 52, FACIT-F scores <30 were reported by 121/485 (24.9%) of patients in LLDAS, 49/240 (20.4%) of patients in DORIS remission, 96/367 (26.2%) of patients attaining sustained LLDAS and 33/178 (18.5%) of patients attaining sustained DORIS remission (figure 1A,B).

Pain/discomfort was the EQ-5D-3L dimension that yielded the greatest frequencies of poor HRQoL experience across the descriptive dimensions of EQ-5D, with responses of ‘some/moderate problems’ or ‘extreme/major problems’ in 156/549 (28.4%) of patients in LLDAS, 75/261 (28.7%) of patients in DORIS remission, 115/408 (28.2%) of sustained LLDAS attainers and 55/197 (27.9%) of sustained DORIS remission attainers (figure 1A,B).

No significant difference was found in the proportion of patients in LLDAS compared with those in DORIS remission or sustained LLDAS versus sustained DORIS remission experiencing poor HRQoL, for any of the target outcomes (online supplemental tables S3 and S4).

### HRQoL outcomes are significantly improved from baseline to week 52 among patients in LLDAS or DORIS remission at week 52

**Figure 3:**
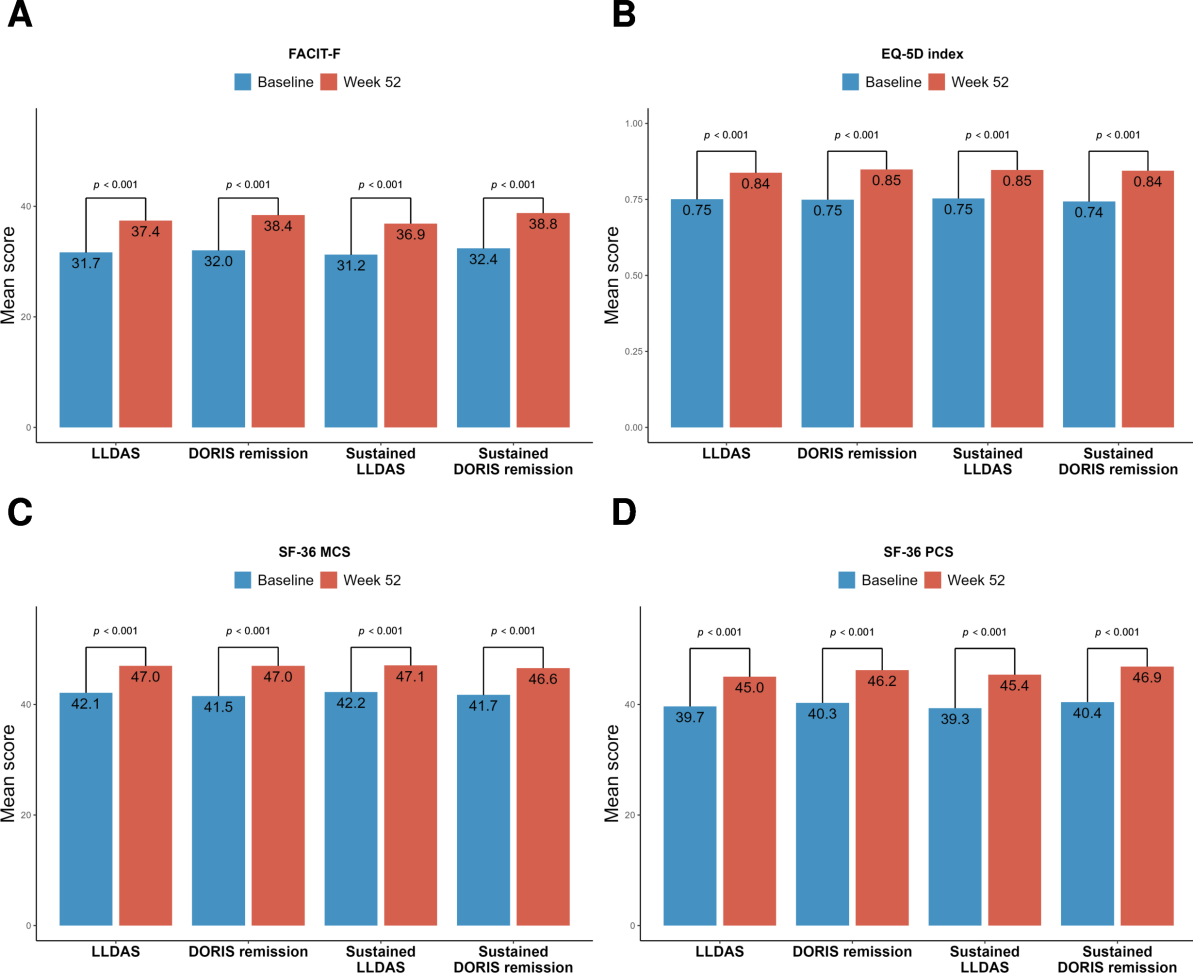
HRQoL outcomes among patients in LLDAS or DORIS remission before and after trial intervention. The bar plots illustrate comparisons between FACIT-F (A), EQ-5D index (B), SF-36 MCS (C) and SF-36 PCS (D) mean scores at baseline (blue bars) and at week 52 (red bars) among patients in LLDAS, DORIS remission, sustained LLDAS or sustained DORIS remission. DORIS, Definition Of Remission in SLE; EQ-5D, EuroQol 5-Dimension health questionnaire; FACIT-F, Functional Assessment of Chronic Illness Therapy – Fatigue; HRQoL, health-related quality of life; LLDAS, Lupus Low Disease Activity State; MCS, mental component summary; PCS, physical component summary; SF-36, short form 36 health survey; SLE, systemic lupus erythematosus.

At week 52 from treatment initiation, patients in LLDAS, DORIS remission, sustained LLDAS or sustained DORIS remission experienced statistically significant and clinically meaningful improvements in all HRQoL outcomes compared with baseline scores, as illustrated in figures3  4 and online supplemental tables S5-S8. FACIT-F mean scores improved from 31.2–32.4 to 36.9–38.8 in the different populations under investigation (p<0.001 for all; figure 3A), as did EQ-5D index mean scores (from 0.74–0.75 to 0.84–0.85; p<0.001 for all; figure 3B). These improvements exceeded the thresholds for MCID.

**Figure 4:**
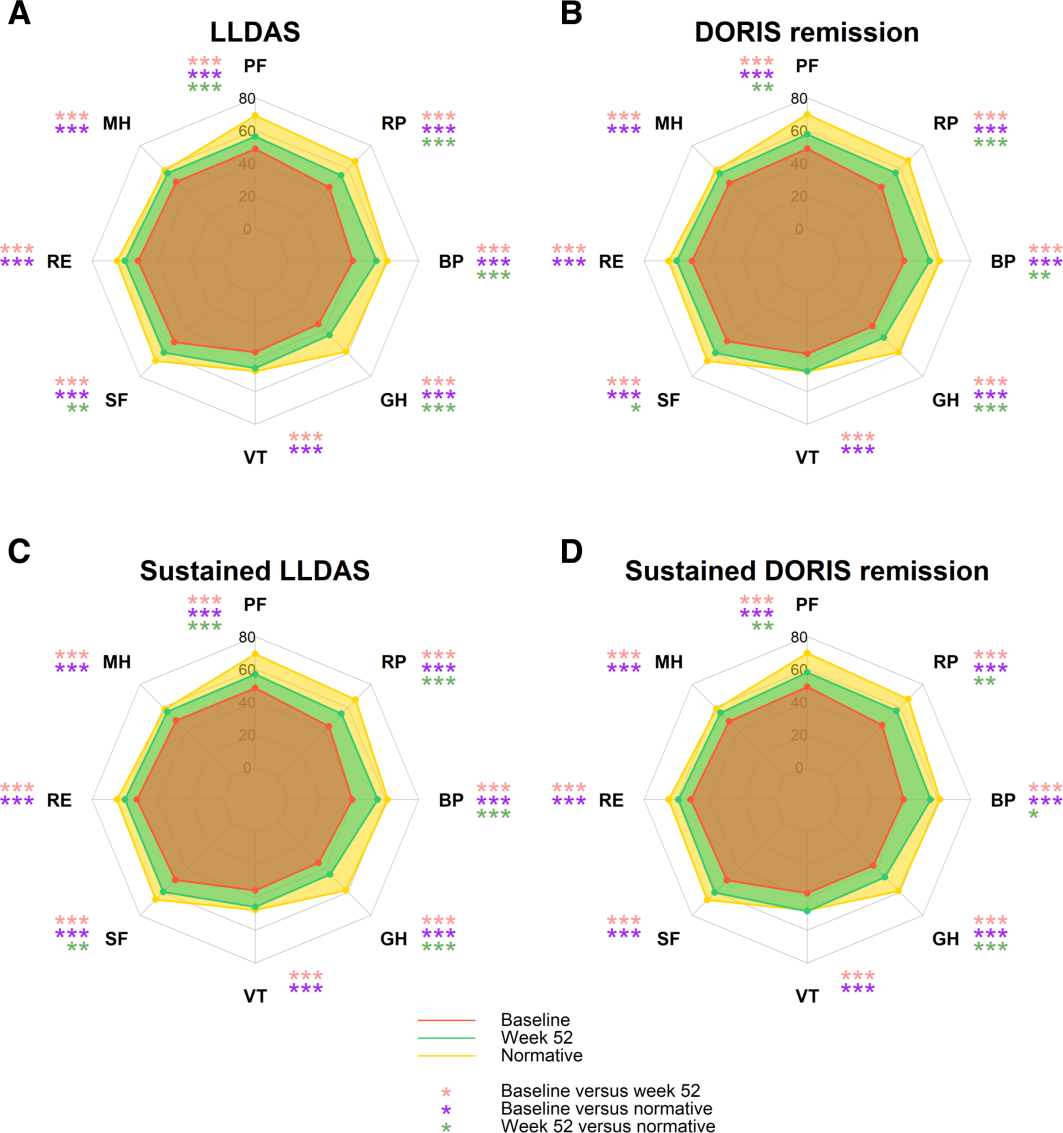
HRQoL outcomes among patients in LLDAS or DORIS remission before and after trial intervention. The radar (spider) charts display mean SF-36 domain scores before (red polygon) versus after (green polygon) trial intervention and versus reference normative values (yellow polygon) among patients in LLDAS (A), DORIS remission (B), sustained LLDAS (C) or sustained DORIS remission (D). Asterisks denote statistically significant differences between baseline and week 52 (orange), between baseline and normative values (purple) and between week 52 and normative values (green). BP, bodily pain; DORIS, Definition Of Remission in SLE; GH, general health; HRQoL, health-related quality of life; LLDAS, Lupus Low Disease Activity State; MH, mental health; PF, physical functioning; RE, role emotional; RP, role physical; SF, social functioning; SF-36, short form 36 health survey; SLE, systemic lupus erythematosus; VT, vitality.

SF-36 MCS mean scores improved from 41.5 to 42.1 at baseline to 46.6–47.0 at week 52 across study population (figure 3C), and SF-PCS mean scores improved from 39.3 to 40.4 to 45.0–46.9 (figure 3D). Those improvements were statistically significant and exceeded the thresholds for MCID (p<0.001 for all comparisons, in all subgroups).

As outlined in figure 4 and detailed in online supplemental tables S9-S12, SF-36 scores at baseline were significantly worse in patients with SLE compared with US population-based norms individually matched for age and sex (p<0.001 for all comparisons, in all patient subgroups). At week 52, all SF-36 component summary and domain scores were significantly increased compared with baseline values. Despite notable improvement, PCS scores remained poorer than US population-based norms for all four patient subgroups (p<0.001 for all), with significant differences observed for all HRQoL domains referred to as physical aspects, that is, PF, RP, BP and GH. On the other hand, MCS scores at week 52 were not significantly different from US population-based norms for any of the four patient subgroups (figure 4 and online supplemental tables S13-S16).

## Discussion

We investigated the frequency of poor HRQoL outcomes in patients with SLE who showed adequate clinical response to therapy, measured using LLDAS and DORIS remission after 1 year of treatment. We showed that even when the therapeutic targets of LDA or remission are achieved, substantial impairments in HRQoL persist, underscoring the discrepancies between physicians’ and patients’ priorities and perception of health state^42^ and the consequent need to address PROs explicitly when defining favourable treatment outcomes.^24 43^

A previous study that investigated the relationship between LDA or remission and HRQoL, the latter assessed using various PRO measures, showed that being in LDA or remission had a positive impact on HRQoL.^20^ In the present study, we observed statistically significant and clinically important improvements in all HRQoL outcomes from baseline after 1 year of trial intervention among patients in LLDAS or DORIS remission. Nevertheless, despite notable improvement compared with baseline, PCS scores at week 52 still were significantly inferior compared with reference normative values for all four patient subgroups studied. Our results align with another post-hoc analysis of the BLISS-52 and BLISS-76 trials, showing that patients with SLE who had met the primary endpoint of the original trials, that is, SLE Responder Index (SRI)-4 response, reported worse mean SF-36 summary component and domain scores at week 52 than US population-based norms.^11^ Overall, proportions of patients in LLDAS/DORIS remission experiencing poor HRQoL outcomes as per the SF-36 health questionnaire were higher within physical vs mental components, with PF (24.0%–26.3%) and GH (16.0%–26.3%) being the SF-36 domains yielding the highest frequencies. This is again consistent with the aforementioned study, in which the same domains yielded the highest frequencies of adverse HRQoL experience among SRI-4 responders.^11^ The observed high frequencies of poor HRQoL outcomes in physical components may partially be explained by the high prevalence of mucocutaneous and musculoskeletal activity in the trial population, which are known to be associated with diminutions in physical HRQoL aspects.^44 45^

Nearly one quarter of patients who were in LLDAS or DORIS remission at week 52 reported FACIT-F scores <30. Similar proportions were found in the pain/discomfort dimension of EQ-5D, with up to 28.7% of patients reporting a response of some/moderate or extreme/major problems. This is in line with observations in other rheumatic diseases; for instance, patients with rheumatoid arthritis have been shown to report considerable frequencies of persisting pain and severe fatigue despite a good clinical response or remission following treatment.^46 47^ Owing to their multifactorial aetiology, inter-individual variability and frequent occurrence in comorbid conditions, pain and fatigue are particularly difficult to assess in clinical practice and are inadequately captured by traditional disease activity indices such as SLEDAI-2K.^43^ Given their high prevalence and debilitating impact in the SLE population,^48^ our findings support the integration of such patient-reported components as relevant treatment targets, complementary to clinical and laboratory measures.

In contrast to prior work focused on response endpoints,^11^ the novelty of the present analysis is that it centres on LLDAS and DORIS remission, which are stringent, state-based targets associated with favourable biological profiles and long-term benefits.^18 19 22^ By quantifying the prevalence of poor HRQoL across multiple PRO instruments among patients meeting these targets and benchmarking against population norms, we show that clinically desirable states do not necessarily translate into acceptable patient-perceived health, particularly for PF, pain and fatigue.

This post hoc analysis is susceptible to selection bias and residual confounding, and trial participants may not fully reflect real-world populations. We lacked data on several determinants known to influence HRQoL, including physician-reported diagnosis of fibromyalgia or depression, socioeconomic context and social support,^43^ precluding comprehensive adjustment. The predefined visit schedule and our sustained-state definition mitigate, but do not eliminate, misclassification of transient states. The 52-week follow-up period may have been insufficient to capture longer-term HRQoL trajectories or benefits of being in LLDAS or DORIS remission over a longer timeframe. Importantly, our analyses were arm-agnostic by design; arm-stratified evaluations within the subset achieving targets were not powered and risk selection bias. Finally, glucocorticoid dose variability was limited by target definitions (≤7.5 mg/day of a prednisone equivalent for LLDAS; ≤5 mg/day of a prednisone equivalent for DORIS remission), constraining inference on glucocorticoid effects; we therefore did not pursue glucocorticoid-focused analyses.

In conclusion, notable frequencies of persistently impaired HRQoL were demonstrated among SLE patients despite low disease activity or remission, indicating that current therapeutic goal definitions do not fully capture patients’ perspectives of health and underscoring the need for more patient-centred targets. While significant improvements in HRQoL were observed over the 52-week treatment period, HRQoL experience in physical domains remained suboptimal compared with population norms. It appears important for future research to prioritise the evaluation of interventions targeting persistent symptoms such as fatigue, pain and emotional distress and aim at developing composite indices that integrate patient-reported health experience along with clinical measures of disease activity.

## Supplementary material

10.1136/rmdopen-2025-006061 online supplemental file 1

## Data Availability

Data are available upon reasonable request.
