# Supplementary material for: Poor health-related quality of life despite Lupus Low Disease Activity State or Definitions of Remission in systemic lupus erythematosus (SLE) remission in patients with SLE: results from a clinical trial setting
Source: RMD Open. 2025 Oct 31;11(4):e006061. doi: 10.1136/rmdopen-2025-006061 (PMC12581079; doi:10.1136/rmdopen-2025-006061)
Supplement: online supplemental file 1 [file rmdopen-11-4-s001.pdf]

Poor health-related quality of life despite LLDAS or DORIS remission in patients with SLE: results from a clinical trial setting

SUPPLEMENTAL MATERIAL

TABLE OF CONTENTS

Supplemental Table S1. Patient demographics and clinical features at baseline. 3

Supplemental Table S2. Proportion of patients experiencing poor HRQoL outcomes despite LLDAS or remission. 5

Supplemental Table S5. Differences in HRQoL outcome scores at baseline compared to week 52 among LLDAS attainers..8

Supplemental Table S6. Differences in HRQoL outcome scores at baseline compared to week 52 among DORIS remission attainers. 9

Supplemental Table S7. Differences in HRQoL outcome scores at baseline compared to week 52 among sustained LLDAS attainers..10

Supplemental Table S8. Differences in HRQoL outcome scores at baseline compared to week 52 among sustained DORIS remission attainers. 11

Supplemental Table S9. Differences in HRQoL outcome scores at baseline compared to normative values among LLDAS attainers...12

Supplemental Table S10. Differences in HRQoL outcome scores at baseline compared to normative values among DORIS remission attainers. 13

Supplemental Table S11. Differences in HRQoL outcome scores at baseline compared to normative values among sustained LLDAS attainers. 14

Supplemental Table S12. Differences in HRQoL outcome scores at baseline compared to normative values among sustained DORIS remission attainers. 15

Supplemental Table S13. Differences in HRQoL outcome scores at week 52 compared to normative values among LLDAS attainers...16

Supplemental Table S14. Differences in HRQoL outcome scores at week 52 compared to normative values among DORIS remission attainers. 17

Supplemental Table S15. Differences in HRQoL outcome scores at week 52 compared to normative values among sustained LLDAS attainers. 18

Supplemental Table S16. Differences in HRQoL outcome scores at 52 compared to normative values among sustained DORIS remission attainers. 19

Supplemental Table S1. Patient demographics and clinical features at baseline.

	All patients (N=2409)	Belimumab + ST (N=1418)	Placebo + ST (N=991)
**Demographics**			
Age* (years); mean (SD)	38.34 (11.82)	38.11 (11.55)	38.67 (12.19)
Female sex; n (%)	2284 (94.8)	1350 (95.2)	934 (94.2)
Race[†]; n (%)			
Asian	426 (17.7)	247 (17.4)	179 (18.1)
White/Caucasian	1032 (42.8)	596 (42.0)	436 (44.0)
Black/African American	632 (26.2)	403 (28.4)	229 (23.1)
Indigenous American[‡]	319 (13.2)	172 (12.1)	147 (14.8)
**Clinical and serological features**			
SLE disease duration (years); mean (SD)[§]	6.56 (6.67)	6.44 (6.65)	6.73 (6.72)
SLEDAI-2K; mean (SD)	10.33 (3.60)	10.40 (3.64)	10.22 (3.55)
<10; n (%)	994 (41.3)	579 (40.8)	415 (41.9)
≥10; n (%)	1415 (58.7)	839 (59.2)	576 (58.1)
PGA score; mean (SD)	1.49 (0.47)	1.50 (0.47)	1.49 (0.47)
0 to 1; n (%)	273 (11.4)	163 (11.5)	110 (11.1)
>1 to 2.5; n (%)	2106 (87.6)	1236 (87.3)	870 (87.9)
>2.5; n (%)	26 (1.1)	16 (1.1)	10 (1.0)
Anti-dsDNA positive[‖]; n (%)	1653 (68.6)	982 (69.3)	671 (67.7)
Low[¶] C3 and/or C4; n (%)	1271 (52.8)	741 (52.3)	530 (53.5)
Low[¶] C3 and/or C4 and anti-dsDNA positive[‖]; n (%)	1088 (45.2)	643 (45.3)	445 (44.9)
aPL positive; n (%)	808 (52.6); N=1536	496 (50.9); N=974	312 (55.5); N=562
**Medications**			
Concomitant SLE medication; n (%)			
GC	2061 (85.6)	1205 (85.0)	856 (86.4)
AMA	1675 (69.5)	981 (69.2)	694 (70.0)
Immunosuppressants	1193 (49.5)	689 (48.6)	504 (50.9)
GC and AMA and immunosuppressants	649 (26.9)	383 (27.0)	266 (26.8)
GC (prednisone equivalent) dose (mg/day); mean (SD)	11.09 (9.04)	11.12 (9.14)	11.05 (8.90)
GC dose (mg/day) category; n (%)			
0	348 (14.4)	213 (15.0)	135 (13.6)
>0 to ≤7.5	637 (26.4)	362 (25.5)	275 (27.7)
>7.5	1424 (59.1)	843 (59.4)	581 (58.6)

Data are presented as numbers (percentage) or means (standard deviation). In case of missing values, numbers of patients with available data are indicated.

*Age was imputed when full date of birth was not available.
[†]Patients who fit more than one race category were counted under the individual race category according to the minority rule as well as the multiracial category.

[‡]Alaska Native or American Indian from North, South or Central America.
[§]Disease duration was defined as (screening date/treatment start date − SLE diagnosis date + 1)/365.25.
[‖]cut-off for anti-dsDNA positivity: ≥30 IU/mL.
[¶]Low C3 cut-off: <90 mg/dL; low C4 cut-off: <10 mg/dL in BLISS-SC, BLISS-NEA and EMBRACE, and <16 mg/dL in BLISS-76 and BLISS-52.
AMA: antimalarial agents; anti-dsDNA: anti double-stranded DNA antibodies; aPL: antiphospholipid antibodies; C3: complement component 3; C4: complement component 4; GC: glucocorticoid; PGA: Physician's Global Assessment; SD: standard deviation; SLE: systemic lupus erythematosus; SLEDAI-2K: SLE Disease Activity Index 2000.

Supplemental Table S2. Proportion of patients experiencing poor HRQoL outcomes despite LLDAS or remission.

HRQoL outcome	LLDAS attainers; n/N (%)	DORIS remission attainers; n/N (%)	Sustained LLDAS attainers; n/N (%)	Sustained DORIS remission attainers; n/N (%)
**SF-36 items**				
PCS ≤ NP5	44/281 (15.7)	19/140 (13.6)	30/210 (14.3)	9/100 (9.0)
MCS ≤ NP5	35/281 (12.5)	16/140 (11.4)	27/210 (12.9)	14/100 (14.0)
PF ≤ NP5	74/281 (26.3)	36/140 (25.7)	51/210 (24.3)	24/100 (24.0)
RP ≤ NP5	20/281 (7.1)	7/140 (5.0)	14/210 (6.7)	4/100 (4.0)
BP ≤ NP5	22/281 (7.8)	10/140 (7.1)	12/210 (5.7)	5/100 (5.0)
GH ≤ NP5	74/281 (26.3)	29/140 (20.7)	55/210 (26.2)	16/100 (16.0)
VT ≤ NP5	27/281 (9.6)	12/140 (8.6)	21/210 (10.0)	8/100 (8.0)
SF ≤ NP5	30/281 (10.7)	17/140 (12.1)	22/210 (10.5)	11/100 (11.0)
RE ≤ NP5	16/281 (5.7)	9/140 (6.4)	12/210 (5.7)	7/100 (7.0)
MH ≤ NP5	23/281 (8.2)	11/140 (7.9)	16/210 (7.6)	10/100 (10.0)
**FACIT-F**				
FACIT-F < 30	121/485 (24.9)	49/240 (20.4)	96/367 (26.2)	33/178 (18.5)
**EQ-5D**				
Mobility level 2 or 3	65/549 (11.8)	24/261 (9.2)	45/408 (11.0)	17/197 (8.6)
Self-care level 2 or 3	23/549 (4.2)	11/261 (4.2)	17/408 (4.2)	8/197 (4.1)
Usual activities level 2 or 3	98/549 (17.9)	38/261 (14.6)	73/408 (17.9)	24/197 (12.2)
Pain/discomfort level 2 or 3	156/549 (28.4)	75/261 (28.7)	115/408 (28.2)	55/197 (27.9)
Anxiety/depression level 2 or 3	89/549 (16.2)	44/261 (16.9)	59/408 (14.5)	33/197 (16.8)

Data are presented as numbers (percentage). BP: bodily pain; DORIS: Definitions Of Remission In SLE; FACIT-F: Functional Assessment of Chronic Illness Therapy – Fatigue; GH: general health; HRQoL: Health-Related Quality of Life; LLDAS: Lupus Low Disease Activity Status; MCS: mental component summary; MH: mental health; NP5: normative 5th percentile; PCS: physical component summary; PF: physical functioning; RE: role emotional; RP: role physical; SF: social functioning; SF-36: short form 36 health survey; VT: vitality.

Supplemental Table S3. Poor HRQoL outcomes in LLDAS attainers compared with DORIS remission attainers.

HRQoL outcome	LLDAS attainers; n/N (%)	DORIS remission attainers; n/N (%)	OR	95% CI	*p* value
**SF-36 items**					
PCS ≤ NP5	44/281 (15.7)	19/140 (13.6)	1.18	0.64–2.24	0.664
MCS ≤ NP5	35/281 (12.5)	16/140 (11.4)	1.10	0.57–2.22	0.874
PF ≤ NP5	74/281 (26.3)	36/140 (25.7)	1.03	0.64–1.69	1.000
RP ≤ NP5	20/281 (7.1)	7/140 (5.0)	1.46	0.57–4.18	0.528
BP ≤ NP5	22/281 (7.8)	10/140 (7.1)	1.10	0.48–2.69	1.000
GH ≤ NP5	74/281 (26.3)	29/140 (20.7)	1.37	0.82–2.32	0.230
VT ≤ NP5	27/281 (9.6)	12/140 (8.6)	1.13	0.53–2.54	0.859
SF ≤ NP5	30/281 (10.7)	17/140 (12.1)	0.86	0.44–1.74	0.743
RE ≤ NP5	16/281 (5.7)	9/140 (6.4)	0.88	0.35–2.32	0.827
MH ≤ NP5	23/281 (8.2)	11/140 (7.9)	1.05	0.47–2.45	1.000
**FACIT-F**					
FACIT-F < 30	121/485 (24.9)	49/240 (20.4)	1.30	0.88–1.93	0.193
**EQ-5D**					
Mobility level 2 or 3	65/549 (11.8)	24/261 (9.2)	1.33	0.80–2.27	0.281
Self-care level 2 or 3	23/549 (4.2)	11/261 (4.2)	0.99	0.46–2.30	1.000
Usual activities level 2 or 3	98/549 (17.9)	38/261 (14.6)	1.28	0.84–1.97	0.269
Pain/discomfort level 2 or 3	156/549 (28.4)	75/261 (28.7)	0.98	0.70–1.39	0.934
Anxiety/depression level 2 or 3	89/549 (16.2)	44/261 (16.9)	0.95	0.63–1.45	0.839

Results from Fisher's exact test comparing poor HRQoL outcome rates between LLDAS and DORIS remission attainers. Data are presented as numbers (percentage), odds ratio (OR), 95% confidence interval (CI), and *p* value. Statistically significant *p* values are in bold. BP: bodily pain; DORIS: Definitions Of Remission In SLE; FACIT-F: Functional Assessment of Chronic Illness Therapy – Fatigue; GH: general health; HRQoL: Health-Related Quality of Life; LLDAS: Lupus Low Disease Activity Status; MCS: mental component summary; MH: mental health; NP5: normative 5th percentile; PCS: physical component summary; PF: physical functioning; RE: role emotional; RP: role physical; SF: social functioning; SF-36: short form 36 health survey; VT: vitality.

Supplemental Table S4. Poor HRQoL outcomes in sustained LLDAS attainers compared with sustained DORIS remission attainers.

HRQoL outcome	Sustained LLDAS attainers; n/N (%)	Sustained DORIS remission attainers; n/N (%)	OR	95% CI	*p* value
**SF-36 items**					
PCS ≤ NP5	30/210 (14.3)	9/100 (9.0)	1.69	0.74–4.21	0.206
MCS ≤ NP5	27/210 (12.9)	14/100 (14.0)	0.91	0.43–1.97	0.858
PF ≤ NP5	51/210 (24.3)	24/100 (24.0)	1.02	0.56–1.86	1.000
RP ≤ NP5	14/210 (6.7)	4/100 (4.0)	1.71	0.52–7.33	0.442
BP ≤ NP5	12/210 (5.7)	5/100 (5.0)	1.15	0.36–4.29	1.000
GH ≤ NP5	55/210 (26.2)	16/100 (16.0)	1.86	0.98–3.70	0.060
VT ≤ NP5	21/210 (10.0)	8/100 (8.0)	1.28	0.52–3.46	0.679
SF ≤ NP5	22/210 (10.5)	11/100 (11.0)	0.95	0.42–2.26	1.000
RE ≤ NP5	12/210 (5.7)	7/100 (7.0)	0.81	0.28–2.50	0.623
MH ≤ NP5	16/210 (7.6)	10/100 (10.0)	0.74	0.30–1.91	0.514
**FACIT-F**					
FACIT-F < 30	96/367 (26.2)	33/178 (18.5)	1.56	0.98–2.51	0.053
**EQ-5D**					
Mobility level 2 or 3	45/408 (11.0)	17/197 (8.6)	1.31	0.71–2.52	0.394
Self-care level 2 or 3	17/408 (4.2)	8/197 (4.1)	1.03	0.41–2.80	1.000
Usual activities level 2 or 3	73/408 (17.9)	24/197 (12.2)	1.57	0.94–2.70	0.077
Pain/discomfort level 2 or 3	115/408 (28.2)	55/197 (27.9)	1.01	0.68–1.51	1.000
Anxiety/depression level 2 or 3	59/408 (14.5)	33/197 (16.8)	0.84	0.52–1.38	0.470

Results from Fisher's exact test comparing poor HRQoL outcome rates between sustained LLDAS and sustained DORIS remission attainers. Data are presented as numbers (percentage), odds ratio (OR), 95% confidence interval (CI), and *p* value. Statistically significant *p* values are in bold. BP: bodily pain; DORIS: Definitions Of Remission In SLE; FACIT-F: Functional Assessment of Chronic Illness Therapy – Fatigue; GH: general health; HRQoL: Health-Related Quality of Life; LLDAS: Lupus Low Disease Activity Status; MCS: mental component summary; MH: mental health; NP5: normative 5th percentile; PCS: physical component summary; PF: physical functioning; RE: role emotional; RP: role physical; SF: social functioning; SF-36: short form 36 health survey; VT: vitality.

Supplemental Table S5. Differences in HRQoL outcome scores at baseline compared to week 52 among LLDAS attainers.

HRQoL outcome	Baseline	Week-52	*p* value
**SF-36 items**			
PF	60.75 (24.66)	70.34 (23.96)	**<0.001**
RP	54.91 (26.29)	67.70 (24.92)	**<0.001**
BP	49.52 (23.10)	67.57 (23.57)	**<0.001**
GH	43.40 (19.39)	55.05 (21.02)	**<0.001**
VT	44.88 (21.84)	57.01 (21.15)	**<0.001**
SF	62.81 (25.39)	74.02 (24.42)	**<0.001**
RE	64.90 (25.79)	74.79 (23.96)	**<0.001**
MH	60.72 (19.88)	70.06 (19.13)	**<0.001**
PCS	39.66 (9.58)	45.03 (9.60)	**<0.001**
MCS	42.12 (11.21)	46.98 (10.53)	**<0.001**
**EQ-5D**			
EQ-5D score	0.75 (0.18)	0.84 (0.16)	**<0.001**
**FACIT-F**			
FACIT-F score	31.65 (11.85)	37.42 (11.06)	**<0.001**

Data are presented as the mean (standard deviation). Statistically significant *p* values are in bold. BP: bodily pain; FACIT-F: Functional Assessment of Chronic Illness Therapy – Fatigue; GH: general health; HRQoL: Health-Related Quality of Life; LLDAS: Lupus Low Disease Activity Status; MCS: mental component summary; MH: mental health; NP5: normative 5th percentile; PCS: physical component summary; PF: physical functioning; RE: role emotional; RP: role physical; SF: social functioning; SF-36: short form 36 health survey; VT: vitality.

Supplemental Table S6. Differences in HRQoL outcome scores at baseline compared to week 52 among DORIS remission attainers.

HRQoL outcome	Baseline	Week-52	*p* value
**SF-36 items**			
PF	61.06 (25.11)	72.15 (24.42)	**<0.001**
RP	55.31 (24.86)	70.54 (23.73)	**<0.001**
BP	49.09 (20.66)	68.66 (23.01)	**<0.001**
GH	45.60 (18.94)	57.80 (20.17)	**<0.001**
VT	45.98 (21.74)	59.51 (20.84)	**<0.001**
SF	61.88 (25.55)	74.46 (24.63)	**<0.001**
RE	63.40 (23.50)	75.18 (24.02)	**<0.001**
MH	59.53 (19.47)	69.92 (19.02)	**<0.001**
PCS	40.29 (9.13)	46.23 (9.12)	**<0.001**
MCS	41.53 (10.72)	46.99 (10.48)	**<0.001**
**EQ-5D**			
EQ-5D score	0.75 (0.17)	0.85 (0.15)	**<0.001**
**FACIT-F**			
FACIT-F score	32.03 (11.89)	38.41 (10.46)	**<0.001**

Data are presented as the mean (standard deviation). Statistically significant *p* values are in bold. BP: bodily pain; DORIS: Definitions Of Remission In SLE; FACIT-F: Functional Assessment of Chronic Illness Therapy – Fatigue; GH: general health; HRQoL: Health-Related Quality of Life; MCS: mental component summary; MH: mental health; NP5: normative 5th percentile; PCS: physical component summary; PF: physical functioning; RE: role emotional; RP: role physical; SF: social functioning; SF-36: short form 36 health survey; VT: vitality.

Supplemental Table S7. Differences in HRQoL outcome scores at baseline compared to week 52 among sustained LLDAS attainers.

HRQoL outcome	Baseline	Week-52	*p* value
**SF-36 items**			
PF	60.59 (23.88)	71.19 (24.14)	**<0.001**
RP	54.55 (25.99)	68.15 (24.57)	**<0.001**
BP	48.97 (23.49)	68.45 (23.10)	**<0.001**
GH	43.13 (19.68)	55.80 (21.46)	**<0.001**
VT	44.34 (21.46)	57.11 (21.67)	**<0.001**
SF	61.90 (25.90)	74.88 (23.42)	**<0.001**
RE	65.77 (26.33)	75.00 (23.85)	**<0.001**
MH	60.94 (20.71)	70.31 (18.95)	**<0.001**
PCS	39.35 (9.58)	45.39 (9.63)	**<0.001**
MCS	42.25 (11.79)	47.06 (10.50)	**<0.001**
**EQ-5D**			
EQ-5D score	0.75 (0.19)	0.85 (0.15)	**<0.001**
**FACIT-F**			
FACIT-F score	31.25 (11.74)	36.88 (11.20)	**<0.001**

Data are presented as the mean (standard deviation). Statistically significant *p* values are in bold. BP: bodily pain; FACIT-F: Functional Assessment of Chronic Illness Therapy – Fatigue; GH: general health; HRQoL: Health-Related Quality of Life; LLDAS: Lupus Low Disease Activity Status; MCS: mental component summary; MH: mental health; NP5: normative 5th percentile; PCS: physical component summary; PF: physical functioning; RE: role emotional; RP: role physical; SF: social functioning; SF-36: short form 36 health survey; VT: vitality.

Supplemental Table S8. Differences in HRQoL outcome scores at baseline compared to week 52 among sustained DORIS remission attainers.

HRQoL outcome	Baseline	Week-52	*p* value
**SF-36 items**			
PF	61.63 (24.01)	72.61 (24.14)	**<0.001**
RP	55.93 (24.64)	71.50 (22.07)	**<0.001**
BP	48.60 (20.41)	69.17 (21.82)	**<0.001**
GH	46.32 (18.91)	58.84 (19.22)	**<0.001**
VT	46.44 (20.50)	60.31 (20.30)	**<0.001**
SF	62.25 (25.62)	75.38 (23.40)	**<0.001**
RE	64.27 (22.60)	73.75 (24.51)	**<0.001**
MH	59.70 (19.07)	69.24 (20.44)	**<0.001**
PCS	40.45 (8.41)	46.86 (7.99)	**<0.001**
MCS	41.73 (10.04)	46.60 (11.03)	**<0.001**
**EQ-5D**			
EQ-5D score	0.74 (0.18)	0.84 (0.15)	**<0.001**
**FACIT-F**			
FACIT-F score	32.39 (11.64)	38.78 (9.99)	**<0.001**

Data are presented as the mean (standard deviation). Statistically significant *p* values are in bold. BP: bodily pain; DORIS: Definitions Of Remission In SLE; FACIT-F: Functional Assessment of Chronic Illness Therapy – Fatigue; GH: general health; HRQoL: Health-Related Quality of Life; MCS: mental component summary; MH: mental health; NP5: normative 5th percentile; PCS: physical component summary; PF: physical functioning; RE: role emotional; RP: role physical; SF: social functioning; SF-36: short form 36 health survey; VT: vitality.

Supplemental Table S9. Differences in HRQoL outcome scores at baseline compared to normative values among LLDAS attainers.

HRQoL outcome	Baseline	Normative values	*p* value
**SF-36 items**			
PF	60.75 (24.66)	86.37 (5.55)	**<0.001**
RP	54.91 (26.29)	83.02 (5.39)	**<0.001**
BP	49.52 (23.10)	76.04 (4.62)	**<0.001**
GH	43.40 (19.39)	73.08 (3.92)	**<0.001**
VT	44.88 (21.84)	59.43 (1.62)	**<0.001**
SF	62.81 (25.39)	82.95 (1.59)	**<0.001**
RE	64.90 (25.79)	80.96 (1.73)	**<0.001**
MH	60.72 (19.88)	73.22 (1.12)	**<0.001**
PCS	39.66 (9.58)	50.95 (2.59)	**<0.001**
MCS	42.12 (11.21)	48.99 (1.03)	**<0.001**

Data are presented as the mean (standard deviation). Statistically significant *p* values are in bold. BP: bodily pain; GH: general health; HRQoL: Health-Related Quality of Life; LLDAS: Lupus Low Disease Activity Status; MCS: mental component summary; MH: mental health; NP5: normative 5th percentile; PCS: physical component summary; PF: physical functioning; RE: role emotional; RP: role physical; SF: social functioning; SF-36: short form 36 health survey; VT: vitality.

Supplemental Table S10. Differences in HRQoL outcome scores at baseline compared to normative values among DORIS remission attainers.

HRQoL outcome	Baseline	Normative values	*p* value
**SF-36 items**			
PF	61.06 (25.11)	87.30 (4.08)	**<0.001**
RP	55.31 (24.86)	83.94 (4.12)	**<0.001**
BP	49.09 (20.66)	76.57 (3.93)	**<0.001**
GH	45.60 (18.94)	73.68 (3.04)	**<0.001**
VT	45.98 (21.74)	59.60 (1.67)	**<0.001**
SF	61.88 (25.55)	83.28 (1.22)	**<0.001**
RE	63.40 (23.50)	81.24 (1.60)	**<0.001**
MH	59.53 (19.47)	73.29 (1.21)	**<0.001**
PCS	40.29 (9.13)	51.32 (2.04)	**<0.001**
MCS	41.53 (10.72)	48.95 (0.99)	**<0.001**

Data are presented as the mean (standard deviation). Statistically significant *p* values are in bold. BP: bodily pain; DORIS: Definitions Of Remission In SLE; GH: general health; HRQoL: Health-Related Quality of Life; MCS: mental component summary; MH: mental health; NP5: normative 5th percentile; PCS: physical component summary; PF: physical functioning; RE: role emotional; RP: role physical; SF: social functioning; SF-36: short form 36 health survey; VT: vitality.

Supplemental Table S11. Differences in HRQoL outcome scores at baseline compared to normative values among sustained LLDAS attainers.

HRQoL outcome	Baseline	Normative values	*p* value
**SF-36 items**			
PF	60.59 (23.88)	86.59 (5.46)	**<0.001**
RP	54.55 (25.99)	83.26 (5.34)	**<0.001**
BP	48.97 (23.49)	76.33 (4.60)	**<0.001**
GH	43.13 (19.68)	73.25 (3.86)	**<0.001**
VT	44.34 (21.46)	59.47 (1.70)	**<0.001**
SF	61.90 (25.90)	83.01 (1.61)	**<0.001**
RE	65.77 (26.33)	81.02 (1.77)	**<0.001**
MH	60.94 (20.71)	73.20 (1.16)	**<0.001**
PCS	39.35 (9.58)	51.07 (2.56)	**<0.001**
MCS	42.25 (11.79)	48.96 (1.04)	**<0.001**

Data are presented as the mean (standard deviation). Statistically significant *p* values are in bold. BP: bodily pain; GH: general health; HRQoL: Health-Related Quality of Life; LLDAS: Lupus Low Disease Activity Status; MCS: mental component summary; MH: mental health; NP5: normative 5th percentile; PCS: physical component summary; PF: physical functioning; RE: role emotional; RP: role physical; SF: social functioning; SF-36: short form 36 health survey; VT: vitality.

Supplemental Table S12. Differences in HRQoL outcome scores at baseline compared to normative values among sustained DORIS remission attainers.

HRQoL outcome	Baseline	Normative values	*p* value
**SF-36 items**			
PF	61.63 (24.01)	87.38 (4.15)	**<0.001**
RP	55.93 (24.64)	83.99 (4.26)	**<0.001**
BP	48.60 (20.41)	76.64 (3.90)	**<0.001**
GH	46.32 (18.91)	73.74 (3.09)	**<0.001**
VT	46.44 (20.50)	59.62 (1.72)	**<0.001**
SF	62.25 (25.62)	83.29 (1.28)	**<0.001**
RE	64.27 (22.60)	81.22 (1.73)	**<0.001**
MH	59.70 (19.07)	73.32 (1.31)	**<0.001**
PCS	40.45 (8.41)	51.36 (2.06)	**<0.001**
MCS	41.73 (10.04)	48.95 (1.04)	**<0.001**

Data are presented as the mean (standard deviation). Statistically significant *p* values are in bold. BP: bodily pain; DORIS: Definitions Of Remission In SLE; GH: general health; HRQoL: Health-Related Quality of Life; MCS: mental component summary; MH: mental health; NP5: normative 5th percentile; PCS: physical component summary; PF: physical functioning; RE: role emotional; RP: role physical; SF: social functioning; SF-36: short form 36 health survey; VT: vitality.

Supplemental Table S13. Differences in HRQoL outcome scores at week 52 compared to normative values among LLDAS attainers.

HRQoL outcome	Week 52	Normative values	*p* value
**SF-36 items**			
PF	70.34 (23.96)	86.37 (5.55)	**<0.001**
RP	67.70 (24.92)	83.02 (5.39)	**<0.001**
BP	67.57 (23.57)	76.04 (4.62)	**<0.001**
GH	55.05 (21.02)	73.08 (3.92)	**<0.001**
VT	57.01 (21.15)	59.43 (1.62)	0.101
SF	74.02 (24.42)	82.95 (1.59)	**0.001**
RE	74.79 (23.96)	80.96 (1.73)	0.124
MH	70.06 (19.13)	73.22 (1.12)	0.712
PCS	45.03 (9.60)	50.95 (2.59)	**<0.001**
MCS	46.98 (10.53)	48.99 (1.03)	0.249

Data are presented as the mean (standard deviation). Statistically significant *p* values are in bold. BP: bodily pain; GH: general health; HRQoL: Health-Related Quality of Life; LLDAS: Lupus Low Disease Activity Status; MCS: mental component summary; MH: mental health; NP5: normative 5th percentile; PCS: physical component summary; PF: physical functioning; RE: role emotional; RP: role physical; SF: social functioning; SF-36: short form 36 health survey; VT: vitality.

Supplemental Table S14. Differences in HRQoL outcome scores at week 52 compared to normative values among DORIS remission attainers.

HRQoL outcome	Week 52	Normative values	*p* value
**SF-36 items**			
PF	72.15 (24.42)	87.30 (4.08)	**0.001**
RP	70.54 (23.73)	83.94 (4.12)	**<0.001**
BP	68.66 (23.01)	76.57 (3.93)	**0.003**
GH	57.80 (20.17)	73.68 (3.04)	**<0.001**
VT	59.51 (20.84)	59.60 (1.67)	0.193
SF	74.46 (24.63)	83.28 (1.22)	**0.035**
RE	75.18 (24.02)	81.24 (1.60)	0.604
MH	69.92 (19.02)	73.29 (1.21)	0.33
PCS	46.23 (9.12)	51.32 (2.04)	**<0.001**
MCS	46.99 (10.48)	48.95 (0.99)	0.668

Data are presented as the mean (standard deviation). Statistically significant *p* values are in bold. BP: bodily pain; DORIS: Definitions Of Remission In SLE; GH: general health; HRQoL: Health-Related Quality of Life; MCS: mental component summary; MH: mental health; NP5: normative 5th percentile; PCS: physical component summary; PF: physical functioning; RE: role emotional; RP: role physical; SF: social functioning; SF-36: short form 36 health survey; VT: vitality.

Supplemental Table S15. Differences in HRQoL outcome scores at week 52 compared to normative values among sustained LLDAS attainers.

HRQoL outcome	Week 52	Normative values	*p* value
**SF-36 items**			
PF	71.19 (24.14)	86.59 (5.46)	**<0.001**
RP	68.15 (24.57)	83.26 (5.34)	**<0.001**
BP	68.45 (23.10)	76.33 (4.60)	**<0.001**
GH	55.80 (21.46)	73.25 (3.86)	**<0.001**
VT	57.11 (21.67)	59.47 (1.70)	0.155
SF	74.88 (23.42)	83.01 (1.61)	**0.009**
RE	75.00 (23.85)	81.02 (1.77)	0.23
MH	70.31 (18.95)	73.20 (1.16)	0.978
PCS	45.39 (9.63)	51.07 (2.56)	**<0.001**
MCS	47.06 (10.50)	48.96 (1.04)	0.345

Data are presented as the mean (standard deviation). Statistically significant *p* values are in bold. BP: bodily pain; GH: general health; HRQoL: Health-Related Quality of Life; LLDAS: Lupus Low Disease Activity Status; MCS: mental component summary; MH: mental health; NP5: normative 5th percentile; PCS: physical component summary; PF: physical functioning; RE: role emotional; RP: role physical; SF: social functioning; SF-36: short form 36 health survey; VT: vitality.

Supplemental Table S16. Differences in HRQoL outcome scores at 52 compared to normative values among sustained DORIS remission attainers.

HRQoL outcome	Week 52	Normative values	*p* value
**SF-36 items**			
PF	72.61 (24.14)	87.38 (4.15)	**0.001**
RP	71.50 (22.07)	83.99 (4.26)	**0.001**
BP	69.17 (21.82)	76.64 (3.90)	**0.011**
GH	58.84 (19.22)	73.74 (3.09)	**<0.001**
VT	60.31 (20.30)	59.62 (1.72)	0.123
SF	75.38 (23.40)	83.29 (1.28)	0.132
RE	73.75 (24.51)	81.22 (1.73)	0.177
MH	69.24 (20.44)	73.32 (1.31)	0.365
PCS	46.86 (7.99)	51.36 (2.06)	**<0.001**
MCS	46.60 (11.03)	48.95 (1.04)	0.626

Data are presented as the mean (standard deviation). Statistically significant *p* values are in bold. BP: bodily pain; DORIS: Definitions Of Remission In SLE; GH: general health; HRQoL: Health-Related Quality of Life; MCS: mental component summary; MH: mental health; NP5: normative 5th percentile; PCS: physical component summary; PF: physical functioning; RE: role emotional; RP: role physical; SF: social functioning; SF-36: short form 36 health survey; VT: vitality.
